# Participating in Online Museum Communities: An Empirical Study of Taiwan’s Undergraduate Students

**DOI:** 10.3389/fpsyg.2020.565075

**Published:** 2021-01-11

**Authors:** Tien-Li Chen, Wei-Chun Lai, Tai-Kuei Yu

**Affiliations:** ^1^Doctoral Program in Design, College of Design, National Taipei University of Technology, Taipei, Taiwan; ^2^Department of Business Administration, National Quemoy University, Kinmen, Taiwan

**Keywords:** emotional resonance, flow theory, social interaction, user involvement, online museum community

## Abstract

With the worldwide spread of the Internet, human activity has become permeated by digital media, which shapes communication and interaction and speeds up the improvement of the experience and diffusion of museum exhibitions. Contemporary museums must understand their audiences, especially with respect to online preferences and surfing involvement experiences. Museums are changing in an effort to attract young netizens to access and use museum resources. Virtual museums are increasingly using digital exhibitions to preserve and apply their collections and establishing online community platforms to interact with young people. This study investigates the underlying mechanism of online community characteristics that enhance audiences’ emotional resonance and involvement. Results from a questionnaire survey (*N* = 1168) of Taiwan undergraduate students show that perceived relevance and esteem improve their emotional resonance, which can attract new people and maintain existing relationships within their communities. Following flow theory, maintaining community relationship characteristics increases emotional resonance, which, in turn, enhances user involvement, but we found only small significant effects of emotional resonance on involvement. These findings illuminate the mechanism of the attitudinal relationship building and maintenance for online museum communities and advance the practical contributions of online museum community use and effects.

## Introduction

Over the last two decades, researchers have attempted to understand and improve several aspects of audience online and off-line experiences, including identifying factors that motivate individuals to visit museums, elements of the visit that influence their overall satisfaction, what is retained following their visit, ways online visitors’ website experiences can be improved, and understanding how all of these combine to encourage visits to museum websites. Museum exhibitions are usually the main channel for education promotion. For museum staff, the main tasks associated with this include understanding more about the online-addiction characteristics of the young, constructing an interesting and interactive museum environment, and increasing involvement in museums (Kabassi, 2017; Roberts and Lyons, 2017). Increasingly, audiences are using social media for a variety of information sharing and online activities (Chi, 2011; Sawyer et al., 2011; Taylor and Gibson, 2017; Pulh et al., 2019). For example, museum audiences understand how to disseminate valuable information and playfulness activities through their own blogs or on social media tools to connect, interact, and share resources with members who have similar interests (Sundar et al., 2015). In the United States, museums have used social media as a marketing channel since the early 2000s, transforming the traditional interaction modes between visitors and exhibits (Roberts and Lyons, 2017). However, although many audiences perceive the value of online communities and expect social media to exhibit a positive impact on formal involvement, the relationships between audiences, social media, and online communities are complex (Csikszentmihalyi et al., 2014; Johnson et al., 2014). It is clear that museum websites serve as important ways to disseminate information. In particular, a growing body of literature is focused on how to encourage online visitors to also visit physical museums to enhance their experience: Increasing online and off-line museum visits is a powerful way to support visitors’ interest and satisfaction (Allen and Crowley, 2014; Siglioccolo et al., 2016; Kabassi, 2017; Falk et al., 2018). Although much attention has been paid to face-to-face interactions with visitors and physical exhibitions within museum settings, little research has focused specifically on online audiences’ museum experiences and management. Because of a lack of benchmark practical cases related to the management of complex museum online communities within digital contexts, additional research is required to better understand how museum online communities can attract online audiences’ interest (Camarero et al., 2018). Museum administrators must weigh the benefits and risks of implementing advanced website and interaction technologies as few museums possess true expertise in the design, development, and maintenance of online communities.

Web 2.0 has redefined the role of museums: hybrid mobile devices allow museums to widen their scope from collections and hardware to digitally generated content and take advantage of Internet-based technology (King et al., 2016; Matuk, 2016; Kabassi, 2017; Roberts and Lyons, 2017). Today, people recognize the importance of digital museum exhibitions in society and making significant contributions to cultural development, education, the content of their physical collections, and overall museum promotion (Russo et al., 2009; Charitonos et al., 2012; Marín-Morales et al., 2019). Museums emphasize direct involvement, personal discovery, and co-creative activities, which encourage interaction with the physical exhibits; this can also use advanced technology to extend to the online environment (Guazzaroni, 2020; Sylaiou et al., 2020). Social media technologies provide new experiences and ways to engage public interest in exhibitions. They offer museum visitors greater cognitive, emotional, and intellectual stimulation, thus changing the way people view, learn, experience, and interpret collections (Dudley, 2017; Falk et al., 2018; Sylaiou et al., 2020). Studies have identified several individual and contextual factors that may influence online communities’ interest, including their prior knowledge and interests, electronic learning materials, digital exhibits and displays, and online social interactions (Csikszentmihalyi and Hermanson, 1995; Dohn, 2011; Nimrod, 2017; Falk et al., 2018; Guazzaroni, 2020). Museum online communities differ from mainstream museums in that their objective comprises both digitizing collections and artifacts and soliciting opinions and building online interactions (Russo et al., 2009; Ch’ng et al., 2019; Guazzaroni, 2020). Online communities may be particularly significant for audiences as they enable interaction and expression of enduring interest through the digital environment (Sylaiou et al., 2010; Sundar et al., 2015; Kim, 2018). This type of online, active engagement plays a critical role in assuring conviviality in real life (Charitonos et al., 2012; Camarero et al., 2019). This observation supplements the considerable evidence of the positive effects of online community involvement on users’ well-being and knowledge growth (King et al., 2016; Morse et al., 2016; Nimrod, 2017).

People engage in museum online communities to make life richer, more wonderful, and more meaningful, but Csikszentmihalyi et al. (2014) observe that enjoyable experiences that make users produce flow have a potentially addictive negative effect. Csikszentmihalyi et al. (2014) note that flow is experienced when individuals fully engage themselves in specific activities, including lack of awareness of time, loss of awareness of the real world, and involvement and a sense of being in the specific environment were flow negative features. Further exacerbating the issue of advanced website and interaction technology risk is the fact that museums are undergoing significant changes in marketing philosophy and online community management. Thus, it is important to consider what types of foundational attraction methods and technologies that the managers prefer (Barker, 2015; Sundar et al., 2015; King et al., 2016; Kim, 2018). Involvement and user experience for visitors are mainly investigated when hybrid reality (including augmented virtuality and reality) or virtual reality technologies play a major role in enabling the connection between visitors and virtual exhibitions (Nah et al., 2011; Pietroni et al., 2018; Ch’ng et al., 2019; Marín-Morales et al., 2019). Social media enables greater interaction and enjoyment as well as joint exploration and discussion between people who are almost all strangers. Knowing how participants perceive the community advantages associated with online museums and the information and experience they can gain during visits can assist managers in determining how to implement online communities of practice (Morse et al., 2016; Dudley, 2017). This research addresses the urgent need of contemporary museums to understand online audiences, specifically regarding their preferences and reactions to website experiences.

Research question 1: We focus on museum visitors participating in the museum online community: How we can make it happen, and how we can manage it to both enhance the online community and evaluate its use?

Research question 2: We attempt to identify the psychological factors that can improve the online interactive design of museum websites and engage audiences, using community involvement as the dependent variable.

## Theoretical Foundations

### Museum Online Communities

Damala et al. (2008) note that, as mobile devices became more powerful, they could be used as new platforms for the interpretative materials to which the visitor is exposed during and after the visit. This, in turn, redefined the relationship between “real” objects and their digital representations: using this two-way communication channel helped to build new online communities out of audiences (Reeve and Woollard, 2016; Lammes, 2017). Geographic location does not constrain participants as physical proximity is not required for bonds to be established: technology creates a new way for communities in which local and global contexts can overlap. Online museum community members are people who love museum collections, and exhibitions shape the way the self is presented, emphasize, or deemphasize certain aspects of their selves to create a desired online impression (Morse et al., 2016; Kim, 2018; Legget, 2018). In user content created worlds, the structure of the online communities can either create or help develop active participation, which allows members to quickly extract interesting, well-organized materials from public information, often leading to the formation of an online community of like-minded individuals (Chi, 2011; Johnson et al., 2014; Siglioccolo et al., 2016; Shin, 2018). In these online communities, participants often ask questions about museum exhibition subjects and are provided with immediate feedback by co-participants, creating a self-initiated and feedback organism (Nah et al., 2011; Ch’ng et al., 2019). Community citizenship typically instructs people to use these social network technologies responsibly to engage in artistic, community, and museum management activities.

According to Hidi and Renninger (2006), people with a developed interest in a subject possess greater knowledge of that subject, value the content more, and are usually more willing to participate in activities than people with an emerging interest. Reviewing the literature, Knogler et al. (2015) indicate that the social interaction between individual and situational interest is positive. Individual interest is consistently rated as an important factor because audiences often have different levels of interest in and knowledge of exhibition subjects. Online communities pursue common values and goals, which depend on continued interactions (Huang et al., 2014; Taylor and Gibson, 2017). These social practices matter in modern societies because they create, hold, and disseminate knowledge and establish competency standards in specific areas (Russo et al., 2009; Sawyer et al., 2011; Jafari et al., 2013; Shaw and Krug, 2013; Lammes, 2017).

### Flow Theory

Subjective experience consists of unique areas of cognition, perception, and emotion, all of which are highly relevant to involvement activity, characterized by positive values and high arousal, and associated with other emotional states, such as happiness, excitement, and ecstasy (Mauri et al., 2011; Weibel et al., 2011; Lin and Utz, 2015). Scholars (e.g., Harvey et al., 1998; Mauri et al., 2011; Barker, 2015; Guo et al., 2016) explain the cognitive and emotional attitudes found in flow theory as proposed by Csikszentmihalyi (1997). His theory refers to individual perceptions during activity participation. According to flow theory, participants “in the flow” exhibit three characteristics: engrossment, believing they can succeed, and immediate feedback. During flow states, participants performing an activity are fully absorbed and feel excitement and enjoyment (Weibel et al., 2011; Csikszentmihalyi et al., 2014; Reeve and Woollard, 2016). Also described as arousal, emotional ease through the interaction between positive emotions and high resonance, participants see high involvement as pleasurable playfulness (Mauri et al., 2011; Guo et al., 2016; Guazzaroni, 2020; Sylaiou et al., 2020).

The flow experience is often described as a steady fade into another state of mind, an extreme mental shift, and even a sense of “losing time,” similar to a dissociative reality state and a loss of self-awareness. According to Csikszentmihalyi (1997), during flow, the emotions are contained and channeled based on the positive, energizing experience associated with the task. Participants who appear to exhibit a flow state note that defining characteristics include the intrinsic need to be engrossed in a fun way and remaining engaged throughout. Csikszentmihalyi et al. (2014) originally defined flow as an emotional experience; during full engagement, people feel that their activity status and emotion are inextricably bound. During a flow situation, museum activity participants are immersed in a feeling of engagement with the activity (Harvey et al., 1998; Morse et al., 2016).

### Hypothesis Development

Museum audiences have new experiences with interactive 2-D or 3-D exhibits through manipulation of motion, sound, and light without the guidance of museum assistants (Reeve and Woollard, 2016; Taylor and Gibson, 2017; Ch’ng et al., 2019; Marín-Morales et al., 2019). Social media technologies seamlessly combine satiation of entertainment gratification and social interaction (Dohn, 2011). Platforms allow users to explore daily and immediate experiences by interacting with different social networks. Interactive strategies can also be created using technology via the multitude of available apps. Relevance relies on one’s ability to assimilate new experiences through the process of interaction. Additionally, expressive individuals often share their knowledge and experience in response to commentary from other members of the community (Brida et al., 2012; Barker, 2015; Sundar et al., 2015; Morse et al., 2016; Pulh et al., 2019). Community members are able to experience relevant topics in the presence of others who seek similar gratification. Online relevance can serve as an alternative or supplement to face-to-face social interactions. Interaction with community members not only involves intrinsic connections, but receiving recognition from others due to legitimate participation promotes greater information sharing within subgroups (Sundar et al., 2015; Le et al., 2016; Siglioccolo et al., 2016; Roberts and Lyons, 2017). Taylor and Gibson (2017) argue that data and information are cold without meaning. Interaction must be cultivated so that knowledge sharing and creation can occur in virtual heritage communities. In many situations, relevance is described by participants as a feeling of being close to others, leading to continued interactions. These relevant relationships lead to increased and persistent emotional resonance (Shaw and Krug, 2013; Legget, 2018; Pulh et al., 2019). In the active dialog pertaining to one exhibition after the exhibition, participants engaged with the ideas of the curator to better understand cultural meanings. In doing so, they became more comfortable with museums and evaluated the museum exhibits as more relevant for life well-being. Based on the foregoing discussion, we hypothesize the following:

H1: Users’ perceived relevance of museum communities is positively related to the members’ emotional resonance.

Ubiquitous access to other community members is a critical success factor provided within the online community for users who are seeking resonance relationships. Chi (2011); Camarero et al. (2018) argue that online community orientation includes socializing, altruism, and building relationships. Users look for more interaction with each other in online communities when they desire long-term relationships. Museums not only connect with the online community via multiple channels, but are also internally structured to facilitate unique interactive experiences that promote the emotional resonance of that community (Springer et al., 2015; Kabassi, 2017). In museum communities, participants through interaction show interest in using simple words and symbols with their connections to generate emotional resonance. Thus, the museum may spend little to maintain its relationships with loyal fans and create resonance for its members. According to Jafari et al. (2013), social interaction is an intrinsic motivating factor in maintaining membership in a community. Other members’ friendships can also fulfill needs. By participating in this interactive sharing of personal opinion, members’ altruism builds collaborative relationships and companionship (Pulh et al., 2019). Le et al. (2016) contend that managers should understand member complexities so that they can cultivate positive altruistic behavior. Therefore, giving online community members greater mutual support provides members with the incentive to continue future interactions. We, thus, construct the following:

H2: Users’ perceived technology interaction with museum communities is positively related to members’ emotional resonance.

A new trend in online museum communities is providing visitors complex or dynamic experiences that encourage them to create conversations involving multiple channels and motivations to find their source of resonance within the space (Ch’ng et al., 2019; Marín-Morales et al., 2019). Experiential activities and forum discussions that occur are often directly or indirectly related to managers’ predesigned exhibition topics, comments, and interactions with other members of the community. Within a community, understanding common codes, graphics, marks, and styles are often seen as part of emotional resonance. Several studies (Brida et al., 2012; Jafari et al., 2013; Manna and Palumbo, 2018) investigate the benefits of esteem and collaborative community partnerships between museums and members of their communities. This process is always dynamic and evolves through established esteem practices and resonance that change over time. Participants may attempt to retain the emotional resonance of a community and their esteem and beliefs within this familiar environment to support and encourage the perpetual online community (Siglioccolo et al., 2016; Camarero et al., 2018). Technological tools and community climates that make individuals feel esteemed make it easier to construct emotional resonance within the community. We, therefore, hypothesize the following:

H3: Users’ perceived esteem of museum communities is positively related to members’ emotional resonance.

Camarero et al. (2018) assert that museums act as generators of creativity, that digital space agency matters, and that those interested in the exhibitions or digital displays can have a common experience with them. The concept of hybridity can be used to foster exhibitions that co-create with the new roles of the museum. The greater the number of participants visiting an exhibit, the more apt they are to communicate, jointly explore and answer questions about exhibits and enjoy the process. Museums can increase immersion and fun with respect to exhibits by combining online communities with social media. A larger number of community members allow for more tactile and tangible forms of interaction and fun with an exhibit (Weibel et al., 2011; Springer et al., 2015; Roberts and Lyons, 2017). The involvement process gives the members direct access to participate in the details of different facets of exhibit experiences. During the experience, participants demonstrate emotional resonance in connection, collaboration, and social interaction with others. Because the social exchanges are unique, they feel like active agents and not passive recipients, delivering added value. Omar et al. (2012) note that the convenience, usefulness, and enjoyment had from interacting with each other on Internet-based media led visitors to communicate more freely and obtain more resonance with the community.

During flow states, participants are often totally immersed in the connection, collaboration, and social interaction experience to the point of losing some sense of self-awareness (Csikszentmihalyi et al., 2014; Shin, 2018). In flow, emotions are positive, energized, and aligned with performing a task. Pierdicca et al. (2019) assert that flow is intrinsically tied to emotional engagement as well as the requirements for task experiences. Participants state that they devote time and effort to their activity because they gain a flow state of experience from it, in which their emotions are positive and energized with full involvement (Shaw and Krug, 2013; Huang et al., 2014; Guo et al., 2016; Chen and Fu, 2018). Flow mediates the connection to the presence and positive levels of emotion, and involvement experience leads to a higher level of resonance (Johnson et al., 2014; Marín-Morales et al., 2019). As discussed in flow theory findings (Weibel et al., 2011; Guo et al., 2016), playfulness activities and positive emotions produce flow, but immersive content or addictive behavior leads to more involvement behavior. Participants who exhibit a positive, playful attitude toward the involved experience are motivated to engage in contextual dialog around virtual exhibits and to continue to experience positive feelings. We, thus, construct Hypotheses 4 and 5:

H4: Users’ perceived playfulness with respect to museum communities is positively related to members’ emotional resonance.
H5: Users’ perceived playfulness with respect to museum communities is positively related to their involvement in museum communities.

Museum diversity gives a community its unique strength, resilience, and richness by identifying members in the community and their interests and unique experiences (Csikszentmihalyi and Hermanson, 1995; Charitonos et al., 2012; Kabassi, 2017; Kim, 2018). In their collections and intellectual property, museums can create effective, unique exhibitions for the public: visitor-centered engagement that visitors can activate based on exhibition uniqueness. Access to online communities provides a unique experience that satisfies users’ curiosity and enables gratification through technological interaction. Camarero et al. (2018) argue that perceiving a sense of a unique citizenship climate within the online community may produce resonance in individuals. Even though participants may perceive museums as presenting their own uniqueness, exhibits cross cultural backgrounds and integrate isolated individuals and communities. Based on real-world social networks and the concept of social capital, increased online involvement may not lead to a decline in real-world engagement. Jafari et al. (2013); Camarero et al. (2018) find that a dependable and like-minded community encourages the use of social media technology tools and community interaction in the physical world. Online communities inspire new ways of museum participation, build uniqueness and brand identity, and make museums more fun. Users receive interpersonal resonance by following micro-leaders and participating more in social relationships (Jafari et al., 2013).

Museum communities must satisfy the needs of all internal and external stakeholders, leading to constantly engaged involvement relationships, depending upon how much effort they devote to it, how much experience they have, and what thoughts are shared. Sylaiou et al. (2010) view involvement as the ways in which an individual’s experience interacts with how they understand the world. However, Derrick et al. (2019) argue that users can satisfy the need for belonging to a community through these social relationships and the involvement of communities. Online museum communities are now collaborative and co-creative platforms without a formal contract: members make voluntary contributions and exhibit automated interactive involvement. Sylaiou et al. (2010); Camarero et al. (2019) observe that online communities represent an interactive component of the museum’s mental and social space in which the museum’s mission or resonance impacts user involvement. The dialog develops when viewers exchange observations, memories, and embodied responses while maintaining an internal dialog as they attempt to create emotional resonance with their interest in the exhibition (Shaw and Krug, 2013; Roberts and Lyons, 2017; Camarero et al., 2018; Legget, 2018). In order for emotional resonance to enhance user involvement, it must become part of an enduring state that involves interest in the exhibits as well as the issues they arouse. As members create resonance via interesting or knowledge-based dialog and receive support for or feedback on their efforts, community knowledge capital increases as do social connections and involvement with other community members. An individual’s engagement behavior with museum audiences is an important topic that has been widely studied in terms of individual attention, emotional resonance, level of learning activities, and affective involvement (Russo et al., 2009; Roberts and Lyons, 2017). In a quantitative study, Roberts and Lyons (2017) use a sociocultural approach during an undergraduate field trip to an art museum to indicate factors that attracted student engagement, such as their prior knowledge, involvement, and hands-on activities. Museum online communities can be viewed as social aggregations in which people have a common resonance, discuss topics publicly, and share points of view about museum activities, leading to personal involvement that exists in cyberspace (Sundar et al., 2015; Pietroni et al., 2018; Sylaiou et al., 2020). We, therefore, hypothesize the following:

H6: Users’ perceptions of the uniqueness of museum communities is positively related to members’ emotional resonance.
H7: Users’ perceptions of the uniqueness of museum communities is positively related to their involvement in museum communities.
H8: Users’ perceptions of the emotional resonance of members is positively related to their involvement in museum communities.

**FIGURE 1:**
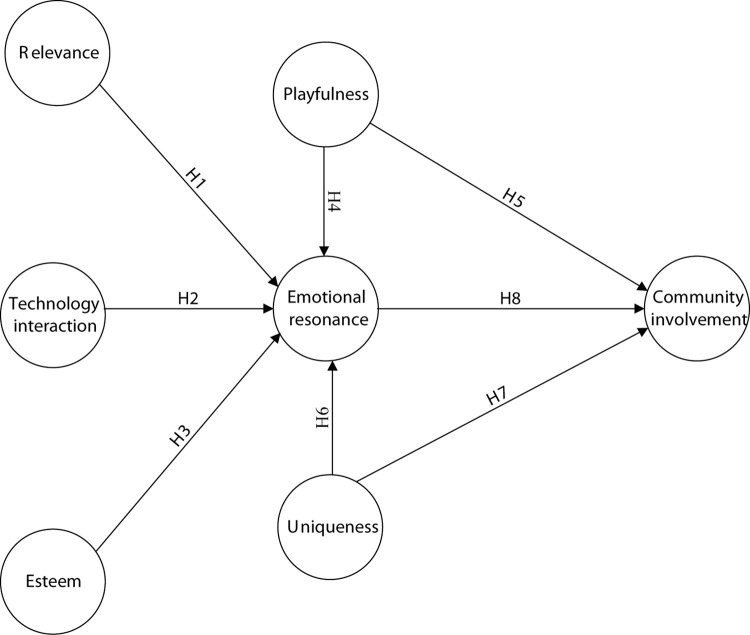
The conceptualized structural research model.

A conceptualized structural model is presented in Figure 1.

## Methods and Descriptive Statistics

The current study constructs a theoretical model that can predict and explain museum community involvement behavior and then empirically tests the model. The validity and reliability of the items were tested to ensure the effectiveness of the research design. A self-administered, closed-ended questionnaire with ordered choices was used to understand undergraduate participation in Taiwan’s museum communities.

### Instrument Measure Development

Using traditional interview approaches and adapted measurement items in online communities, we attempt to understand the visitor experience in museum online communities through visitors’ behavior and preferences. The flow theory literature provides a comprehensive foundational perspective on consciousness and perception and integrates the various components of online museum communities as we construct the research model. Our research model combines flow theory and information communication technology to uncover the psychological factors that influence individuals’ involvement behavior. Three independent constructs were identified from flow theory and ICT interaction: playfulness, uniqueness, and technology interaction. A total of 17 questionnaire items for these three constructs were adapted from those in Mauri et al. (2011), Jafari et al. (2013), Shaw and Krug (2013), Csikszentmihalyi et al. (2014), Guo et al. (2016), King et al. (2016), Camarero et al. (2018), Kim (2018). Member perceptions of esteem and relevance in online communities, which include captivation and intangible experiences, relate to the social media tools used to enable the creation of immersion, flow, and physical pleasure pertaining to online community environment (Barker, 2015; Le et al., 2016; Legget, 2018). Given this, scholars contend that, only when individuals perceive esteem and relevance from a community will they habitually place the online community as a top priority. Because esteem and relevance within the community climate are intangible, the present study modifies items drawn from the survey approach of Barker (2015), which measures direct sensory perception, along with Siglioccolo et al. (2016), Legget (2018), constructing a total of six measurement items.

According to research, triggered situational interest is a temporary psychological state that describes the arousal of a person’s attention for a short period of time by affect, novelty, or external information, in turn creating more enduring and persistent individual interest (Hidi and Renninger, 2006; Wigfield and Cambria, 2010; Knogler et al., 2015; Falk et al., 2018). Following the definition of Hidi and Renninger (2006); Knogler et al. (2015), interest is a protracted tendency to engage with a particular subject or activity over time. The interest questionnaire used in the current study was adapted from Knogler et al. (2015) and includes two dimensions: emotional attention or affect and continual involvement in related tasks. Therefore, in this study, audience emotional resonance was conceptualized as an affective construct that represents the positive emotion and enjoyment that audiences experience during their participation. The term “involvement” as viewed from a participant’s perspective can be defined as easily interacting with community members, knowledge sharing and constructing, and switching between friendly online and off-line environments (Pietroni et al., 2018). To mitigate difficulties in measuring and observing continual participate behaviors, the present study used emotional resonance and involvement as indicators for continual behaviors, modifying question items from Sawyer et al. (2011); Charitonos et al. (2012), Knogler et al. (2015); Pietroni et al. (2018), Ch’ng et al. (2019) and on emotional resonance (four items) and community involvement (six items), and statements were modified to fit the context of the museum’s online community.

To operationalize this study, we converted each of the constructs into statements that could be measured using Likert scales. All items were measured using a 5-point Likert-type scale ranging from “strongly agree” to “strongly disagree” (see Appendix Table A1 for survey items). These statements were included in our questionnaire to evaluate users’ subjective insights into experiences with museum websites and communities. Two stages were conducted in the pretest. Participants were allowed to complete the questionnaire on their own in 20 min, but could seek clarifications from the researchers at any time during the process in the first stage. After completing the questionnaires, researchers explained meaning of every question to participants and ensured no misinterpretation. The questionnaire was modified to ensure all statements were unambiguous and that participants would not feel overly burdened while completing it. This method allowed each participant to focus on latent insights while creating a compact instrument and avoided possible sources of misunderstanding. The initial questionnaires were administered to 100 students who reported that they had participated in a museum’s online community. The pilot test Cronbach’s α reliability scores ranged from 0.795 for relevance to 0.935 for playfulness, implying that the scales used in this study satisfactorily measured the constructs of interest.

### Sample and Descriptive Statistics

Data collection in this study consisted of two parts: a face-to-face quantitative questionnaire and online survey. Participation was completely voluntary. Participants had to be 18 or older and have experience interacting with museum communities via the Internet or mobile applications. On the survey, respondents identified their absolute usage of the museum online community or digital exhibitions, and then invitation e-mails were sent to potential participants to answer a series of questions on an attached URL linked to a web-based survey form. The face-to-face survey was conducted from April 1, 2018, to June 19, 2018, in six universities. Responses submitted by the end of the 60th day after the survey request were used in this study. A total of 1500 questionnaires were sent out. Participants who interacted less than twice within a 6-month period were excluded. After 2 months, a total of 1300 questionnaires were returned: 132 of these were considered problematic because of excessive missing data, “don’t know,” or N/A answers and response bias (e.g., responses were all 3 or 5 on the scale, outliers, and missing data). After filtering, 1168 valid responses (89.85%) were retained for analysis. The sample demographic data in Table 1 depicts a diverse cross-section of the population: 30.8% were male, and 69.2% were female. Their average age was 21.5 years (standard deviation = 3.31). Participant information resources varied: social media was 50.2%, advertisements were 40.2%, public relations or news reports were 39.6%.

**TABLE 1:** Respondent profiles.

**Demographics**	**Level**	**Count**	**Percentage**
Gender	Male	360	30.8
	Female	808	69.2
Age	Average	21.50	
	Maximum	57	
	Minimum	18	
	Missing value	7	
Information resources	Advertisement	491	42.0
	Public relation or news report	463	39.6
	Use keyword	188	16.1
	Promotion method	106	9.1
	Use the public media	157	13.4
	Official event	246	21.1
	Social media	607	52.0
	Community or nonprofit organization	141	12.1
	Websites	157	13.4
	Volunteer	30	2.6
	Other foundations	6	0.5
	Handbooks	21	1.8
	Special issue	42	3.6
	Direct marketing	11	0.9
	Cooperation manufacturer	31	2.7
	Sponsor	17	1.5
	Promotion of leaflet	129	11.0

## Results

### Measurement Model Evaluation

We used SmartPLS3.0 (developed by Ringle et al., 2015) to analyze the data, evaluating convergent and discriminant validity and using a resampling bootstrapping process to obtain standard error rates and *t*-values. Based on the suggestions of Bagozzi and Yi (1988), we chose three of the most common indicators to assess the validity of the measurement model. First, we examined the measured variables’ factor loadings on loading values. A rule of thumb is that factor loadings must exceed 0.7 within exploratory research contexts. Second, because using Cronbach’s α to evaluate the reliability of constructs would underestimate their values, we opted for composite reliability (CR) (Bagozzi, 2011). All constructs were deemed reliable as they all possessed Cronbach’s α figures that exceeded 0.803. Fornell and Larcker (1981) recommend that CR values should be greater than 0.6. In this study, CR values ranged from 0.884 to 0.925, exceeding the 0.7 threshold, thereby indicating favorable internal consistency (Table 2). Third, to test the discriminant validity, the value of average variance extracted (AVE) must exceed 0.5 (Fornell and Larcker, 1981). The AVE figures for latent variables in this study ranged from 0.630 to 0.729. The questionnaire items were also tested using the correlation matrix and for discriminate power to ensure their reliability and validity.

**TABLE 2:** Reliability and validity indicators of the research model.

**Construct**	**Cronbach’s Alpha**	**rho_A**	**Composite Reliability**	**Average Variance Extracted (AVE)**
Esteem	0.803	0.810	0.884	0.719
Technology interaction	0.855	0.867	0.895	0.630
Relevance	0.806	0.812	0.885	0.720
Uniqueness	0.873	0.874	0.908	0.664
Playfulness	0.905	0.911	0.925	0.640
Emotional resonance	0.875	0.882	0.915	0.729
Community involvement	0.898	0.899	0.922	0.663

Following Fornell and Larcker (1981), we evaluated the discriminant validity of the research model to determine whether the square root of each construct’s AVE was greater than the correlation between that construct and any other within the model. As shown in Table 3, the constructs’ intercorrelation coefficients ranged from 0.535 to 0.802. Thus, the items overall demonstrated satisfactory convergent and discriminant validity.

**TABLE 3:** The discriminant validity of research model.

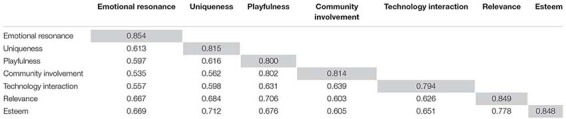

*All correlations are significant at the *0.001* level; diagonal element is square root of AVE and should be larger than the off-diagonal correlation coefficient.*

### Testing the Structural Model

**FIGURE 2:**
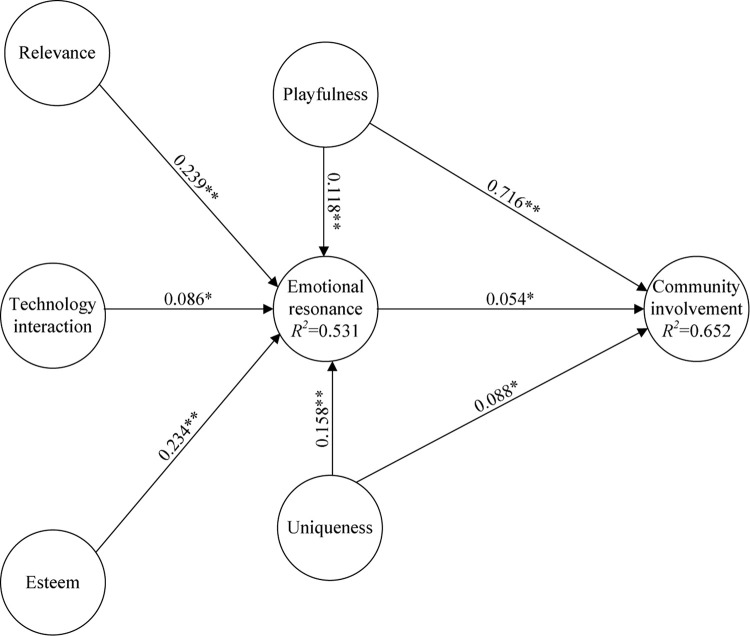
Path coefficients for the research model. ^∗^: <0.05; ^∗∗^: <0.01.

“A structural model is analyzed to investigate and depict the link among variables in a proposed model” (Byrne, 1998). To estimate the significance of the path coefficients and test the hypotheses, a bootstrapping method using 5000 bootstrap subsamples was conducted in SmartPLS 3.0. The standardized path coefficients represent direct effects, path coefficients, *t*-values, and *p*-values as shown in Table 4. All the path coefficients were significant (*p* < 0.05) with *t*-values larger than 1.96. These paths reflect the impact of relevance (H1), technology interaction (H2), esteem (H3), playfulness (H4), and uniqueness (H6) on emotional resonance (0.239, 0.086, 0.234, 0.118, and 0.158) and playfulness (H5), uniqueness (H7), and emotional resonance (H8) on community involvement (0.716, 0.088, and 0.054, respectively). Path coefficients for each value from the models are shown in Figure 2. They confirm that the model explains a substantial portion of the variance in all the endogenous variables: 53.1% for emotional resonance and 65.2% for community involvement. The explanatory power of the endogenous latent variables approaches or exceeds the recommended value of 0.5, suggesting that the proposed research model was robust and stable.

**TABLE 4:** Paths coefficient and *t*-value.

**Path**	**Beta**	**Mean**	**Std Dev**	***t*-Value**	***p*-Values**
Relevance- -> Emotional resonance	0.239	0.239	0.043	5.614	0.000
Technology interaction- -> Emotional resonance	0.086	0.087	0.039	2.167	0.030
Esteem - -> Emotional resonance	0.234	0.234	0.049	4.812	0.000
Playfulness- -> Emotional resonance	0.118	0.119	0.040	2.931	0.003
Playfulness- -> Community involvement	0.716	0.716	0.024	29.959	0.000
Uniqueness- -> Emotional resonance	0.158	0.158	0.037	4.314	0.000
Uniqueness- -> Community involvement	0.088	0.087	0.029	3.060	0.002
Emotional resonance- -> Community involvement	0.054	0.055	0.027	2.037	0.042

Next, the direct effects of antecedents on the flow of emotional resonance (i.e., relevance, technology interaction, esteem, playfulness, and uniqueness) were examined. Online communities become relevant to participants because they offer a feeling that a given community matches participants’ views of the world and allows them to pursue their interests and concerns (Shaw and Krug, 2013; Sundar et al., 2015; Siglioccolo et al., 2016). The use of online communities certainly increases the accessibility of information and speed of communication. It also increases resonance with the museum’s exhibits and involvement in immersion activities. Jafari et al. (2013) establish an interactive sociality strategy for museums and note that social and interactive technology in online or off-line environments can allow members to feel esteem and establish relevance in the community. Respondents emphasized a process of communication that requires the continual exchange of thoughts and feelings, generating engaged discussion in the online community. Likewise, Brida et al. (2012), Le et al. (2016) find that esteem affects members’ willingness to stay or leave and positively impacts the resonance of the community. In the online community, in addition to the members’ understanding of the museum’s purpose, for pursuit of knowledge growth and cultural literacy improvement, meaningful and pleasant memories of community activities are important in establishing relevance for the community. Following Camarero et al.’s (2018) basic principles, guiding emotional resonance is the notion that individuals will strategically select media to satisfy their entertainment and social-psychological needs. In short, members have external incentives (relevance, technology interaction, esteem) and belief incentives of exhibition content (playfulness, uniqueness) with the emotional resonance of the museum community as an intermediary to satisfy their exhibition visitation needs.

Several studies (Nah et al., 2011; Sawyer et al., 2011; Omar et al., 2012; Kim, 2018) employing the flow theory in online communities report playfulness to be positively correlated with emotional resonance and immersion involvement. The emotional resonance experience is grounded in a perceiver’s own connection to past positive or negative emotions and community experiences. Such experiences are not necessarily the same for different people or at different communities. In a virtual museum, members can automatically make connections between exhibits and their own online inquiries, issues, and interests, enabling learning to become deeply motivating and meaningful (Springer et al., 2015). In many cases, online communities are given the opportunity to access revisited content (e.g., exhibition stories and streams) and increase their emotional resonance and immersion involvement, which can propel exhibitions into the limelight temporarily or quickly. In the online community, the higher the emotional resonance generated by the theme exhibition of the museum, the more members will actively interact and share, and the greater the immersive involvement of the community (Siglioccolo et al., 2016; Pietroni et al., 2018; Sylaiou et al., 2020). Member develop their own unique meaning for exhibitions or objects, and these meanings and values can change together with interaction resonance or the values associated with audiences’ immersion experiences.

## Discussion and Limitations

### Discussion

This study examines the relationship between institutions and online communities and clarifies the engaged forms of a diverse audience. Social media combined with forums serve as a central tool to connect users with physical museum exhibitions, where physical and online experiences are linked in captivating ways that create stories and extended knowledge for visitors (Sundar et al., 2015; Kim, 2018; Pietroni et al., 2018; Shin, 2018; Ch’ng et al., 2019). In this sense, the importance of interactive playfulness should be emphasized because playfulness is positively associated with emotional resonance and involvement. Online museum communities should act as a catalyst for promoting new learning based on the interests and previous knowledge of individuals. As platforms of knowledge and cultural representation, online communities can act as catalysts for museum change by building bridges between professional interest groups and across society (Brida et al., 2012; Charitonos et al., 2012; Sundar et al., 2015; Morse et al., 2016; Kim, 2018), embedding community opinions in the meaning and sense of exhibitions, cultivating awareness of social roles, and increasing interest in the museum through entertaining components that engage those with whom they co-create. Based on the results of this study, online museum community managers should encourage participants to form their own online teams based on existing resonances to maximize the potential for emotional arousal and its benefits for engagement. This concept echoes the conclusions of Charitonos et al. (2012); King et al. (2016), Dudley (2017); Camarero et al. (2018), Pierdicca et al. (2019). Online exhibitions generate a distinctive, vibrant museum resonance experience whose purpose is to enhance the users’ online involvement through its attributes of interactiveness and playfulness. Through social media platforms, museums can respond to user opinions and suggestions by re-contextualizing their collections. This can promote citizens’ interest in cultural artifacts as well as museum online community involvement.

Meanwhile, we identify relevance and esteem as means to increase individual emotional resonance, consistent with previous studies (Sundar et al., 2015; Roberts and Lyons, 2017; Taylor and Gibson, 2017; Kim, 2018; Derrick et al., 2019). This allows the manager to respect how participants care about what topics and how they can make meaningful choices based on interactive experiences. Community members provide dialog and knowledge resonance as inputs to the process through which the visitors and the museum service providers become coproducers of experiences. Our findings are conducive to the social interaction construction core of emotional resonance. Audiences expect an immersive experience when they visit a museum, but this experience gap is eliminated when social media is integrated with online and off-line experiences that are intended to promote connections between visitors concerning themes that are personally relevant and interesting to them. Social media can help overcome limitations associated with exhibition interpretations by providing current interactive content without the need for renovating a physical dialog channel. Museum staff must understand how instructional technology interaction design and development are specifically applied to users’ online experience in a museum community context, which can satisfy individual needs for relevance and esteem.

Many museums have recently undergone enormous changes in their digital exhibition and social interaction programs, which highlights the potential of enhanced playfulness and uniqueness (Russo et al., 2009; Dohn, 2011; Sawyer et al., 2011; Shaw and Krug, 2013; Sundar et al., 2015; Roberts and Lyons, 2017). Playful activities that produce flow are not only pleasurable, but also easily become addictive as they have the potential to enhance unique meaning and life. The depth of esthetic feeling, curiosity, and meaning-sense activities are related to cognitive and playfulness motives and can provide the museum’s audience with additional information to generate emotional resonance or increase involvement. This is consistent with the conclusions of Harvey et al. (1998); Csikszentmihalyi et al. (2014), Springer et al. (2015); Guazzaroni (2020). To engage participants, online museum communities have used augmented reality, virtual reality, and mixed reality technologies to extend museum concepts beyond physical limits, create sensory experiences, and reduce institutional barriers. When a museum manager clearly directs a discussion to exhibit themes, members who encounter these themes are more likely to achieve leisure and entertainment and receive feedback. The expanding digital mission of museums reflects their internal awareness and also serves as a way to attract online community visitors (Sawyer et al., 2011; Lammes, 2017; Falk et al., 2018; Pierdicca et al., 2019).

Finally, online communities have synthesized cognitive, sociological, and esthetic approaches to understanding museum collections in an effort to increase interactive involvement. Engaging in esthetic activities can catalyze personal growth and transformation. While appreciating exhibits, consistent with the literature, discussion of multiple meanings and exchanging thematic ideas is important as there are multiple co-creative interpretations and knowledge that can be constructed (Harvey et al., 1998; Jafari et al., 2013; Allen and Crowley, 2014; Kim, 2018; Derrick et al., 2019). Participants in this study mentioned they better appreciated exhibits due to the additional interaction and involvement available through social communication technology. The active museum’s online communities played a facilitator role in those visitors’ online experiences, which brought about a greater focus and engagement (Chi, 2011; Sawyer et al., 2011; Barker, 2015; Taylor and Gibson, 2017; Derrick et al., 2019; Pierdicca et al., 2019). Museum online community and discussion forums allow participants to express different perspectives about the same exhibition and listen to each other. In addition to feelings of esteem and relevance associated with their engagement with the exhibition content, a significant number of participants agreed that they had experienced an emotional resonance and said that they felt more committed to involvement in the online community going forward.

### Limitations

Virtual museums allow members to choose the depth of their engagement with exhibits through nuanced digital presentations that appeal to diverse audiences. This study used a limited test sample to measure personal presence in relation to a specific online interactive experience. With the self-reported questionnaire, there is risk of a response bias for participants from different sources. Before the data from the online and off-line sources of respondents were combined into PLS analysis, we suggest that chi-square or parameter statistical analysis be used to investigate the differences between the data collected from off-line and online sources. However, we did not further analyze or explore the relationship between digital exhibition types and involvement behavior. These results may be specific to a given online community, which may limit generalization of the research findings to other populations. Additional investigations with other types of exhibitions are necessary to generate findings that are more robust and generalizable. We did not specifically separate the participants into high and low emotional resonance groups to test model differences. Future researchers should consider this. Future studies can explore the characteristics of flow theory and online community camaraderie principles to learn more about participant interactions with other participants as well as with the online community system.

## Conclusion

In 2019, the Taiwan government’s Ministry of Culture proposed a draft “Cultural Science and Technology Act” to encourage museums to use technology for digital applications and promote cultural participation and friendly access use to help shape the cultural civil society of the digital age. Growth in culture tourism has prompted the construction of new museums and the adjustment of existing exhibits to include more complex knowledge and new media. Major museum-related exhibitions and events are dedicated to communication and interaction within online communities. Online museum communities are unique and important: Internet-based communication allows for the discussion of enhanced experiences and objectives as compared to traditional museums. Our results indicate that perceived uniqueness marginally increases emotional resonance but also slightly improves user involvement. Audience perceived playfulness is a powerful way to increase user involvement, and emotional resonance has a weak but significant effect on user involvement. Interactivity between members and exhibit resonance motivate audiences to become involved in online communities. Such communities are important for museums. Perceived relevance and esteem marginally increase emotional resonance, which enhances user involvement. These findings provide important insights for researchers and professionals into the significant role played by museum managers as they engage audiences through providing interactive experiences in online environments.

## Data Availability Statement

The original contributions presented in the study are included in the article/supplementary material, further inquiries can be directed to the corresponding author/s.

## Ethics Statement

Ethical review and approval was not required for the study on human participants in accordance with the local legislation and institutional requirements. Written informed consent to participate in this study was provided by the participants’ legal guardian/next of kin.

## Author Contributions

T-LC contributed to concept and design, interpretation of data, and writing up. W-CL contributed to data collection, interpretation of data, writing up, and study supervision. T-KY contributed to concept and design, data collection, statistical analysis, interpretation of data, and writing up. All authors wrote the manuscript together and approved the final manuscript.

## Conflict of Interest

The authors declare that the research was conducted in the absence of any commercial or financial relationships that could be construed as a potential conflict of interest.

## Appendix A

**TABLE A1:** Questionnaire survey items.

**Construct**	**Measurement item**	**Mean**	**S.D.**
Esteem	This website is very popular among visitors.	3.798	1.189
	I will proudly say that I have joined this museum’s community.	3.628	1.182
	The popularity and recognition of the community are higher than that of other museums.	3.680	1.159
Technology interaction	This website uses the activity viewing board and audience interaction to guide visits.	3.622	1.099
	This website uses a wireless guidance system (APP or QR code) to guide visitors.	3.440	1.118
	This museum uses an automated touchscreen audiovisual navigation system to guide visitors.	3.371	1.111
	This community provides space for social interactions.	3.665	1.106
	This community provides related written information for visitors to read.	3.607	1.099
Relevance	This community provides more meaning to the artistic lives of visitors.	3.762	1.142
	This community can fill visitors with happy memories.	3.774	1.187
	This community easily reminds visitors of its products and services.	3.528	1.069
Uniqueness	The appearance of this community is very different from other museums.	3.627	1.204
	The local features of the community present very differently from that of other museums.	3.646	1.123
	Promotion of the community is presented very differently from that of other museums.	3.528	1.073
	Attributes of online exhibition of the community are very different from that of other museums.	3.572	1.048
	Attributes of online activities of this community are very different from that of other museums.	3.537	1.033
Playfulness	I feel this community provides visitors with a fun experience.	3.509	1.066
	I feel this community is very esthetically pleasing.	3.456	1.021
	I feel that this community allows me to control the combination of layout and content.	3.248	1.028
	I feel this community can stimulate visitors’ curiosity.	3.520	1.036
	I feel this community meets the needs of the visitors.	3.532	1.030
	I feel the community provides meaningful interactions with visitors.	3.520	1.040
	I feel the community highlights interesting information on visitors’ participation in activities.	3.505	1.040
Emotional resonance	I am interested in knowing more about this museum’s activities through the community.	3.574	1.099
	When I see discussions about the museum’s community on social media, I am interested.	3.517	1.120
	Compared to other people, I am more interested in this community’s news.	3.316	1.071
	If this museum’s community shuts down, I will miss it.	3.593	1.228
Community involvement	The museum’s community allows visitors or volunteers to form a community.	3.432	1.032
	The museum community provides the fruits of participants’ communication.	3.397	1.010
	The museum community encourages close interaction among participants.	3.431	1.004
	The museum community’s interactions revolve around an interesting theme.	3.493	1.055
	Autolinks can be generated on the community when members share on Facebook, Instagram, or LINE.	3.435	1.047
	The museum’s community uses themed activities to support online or offline relationship maintenance.	3.418	0.967
